# Influence of *GSTM1*, *GSTT1*, and *GSTP1* Polymorphisms on Type 2 Diabetes Mellitus and Diabetic Sensorimotor Peripheral Neuropathy Risk

**DOI:** 10.1155/2015/638693

**Published:** 2015-09-08

**Authors:** Adina Stoian, Claudia Bănescu, Rodica Ioana Bălaşa, Anca Moţăţăianu, Mircea Stoian, Valeriu G. Moldovan, Septimiu Voidăzan, Minodora Dobreanu

**Affiliations:** ^1^Department of Pathophysiology, University of Medicine and Pharmacy, Gheorghe Marinescu 38, 540139 Tîrgu Mureş, Romania; ^2^Department of Medical Genetics, University of Medicine and Pharmacy, Gheorghe Marinescu 38, 540139 Tîrgu Mureş, Romania; ^3^Department of Neurology, University of Medicine and Pharmacy, Gheorghe Marinescu 38, 540139 Tîrgu Mureş, Romania; ^4^Intensive Care Unit, Mureș County Hospital, Bernády György 6, 540000 Tîrgu Mureş, Romania; ^5^Department of Epidemiology, University of Medicine and Pharmacy, Gheorghe Marinescu 38, 540139 Tîrgu Mureş, Romania; ^6^Department of Laboratory Medicine, University of Medicine and Pharmacy, Gheorghe Marinescu 38, 540139 Tîrgu Mureş, Romania

## Abstract

*Background and Aims*. Diabetic neuropathy is a frequent complication of type 2 diabetes mellitus (T2DM). Genetic susceptibility and oxidative stress may play a role in the appearance of T2DM and diabetic neuropathy. We investigated the relation between polymorphism in genes related to oxidative stress such as *GSTM1*, *GSTT1*, and *GSTP1* and the presence of T2DM and diabetic neuropathy (DN). *Methods*. Samples were collected from 84 patients with T2DM (42 patients with DN and 42 patients without DN) and 98 healthy controls and genotyped by using polymerase chain reaction and restriction fragment length polymorphism method. *Results*. *GSTP1* Ile105Val polymorphism was associated with the risk of developing T2DM (*p* = 0.05) but not with the risk of developing DN in diabetic cases. *GSTM1* and *GSTT1* gene polymorphisms were associated with neither the risk of developing T2DM nor the risk of DN occurrence in diabetic patients. No association was observed between the patients with T2DM and DSPN (diabetic sensorimotor peripheral neuropathy) and T2DM without DSPN regarding investigated polymorphism. *Conclusion*. Our data suggest that *GSTP1* gene polymorphisms may contribute to the development of T2DM in Romanian population. *GSTM1*, *GSTT1*, and *GSTP1* gene polymorphisms are not associated with susceptibility of developing diabetic neuropathy in T2DM patients.

## 1. Introduction

Diabetes mellitus is a global health problem affecting children, adolescents, and adults occurring when the pancreas does not produce enough insulin or/and when the body cannot effectively use the insulin produced by the pancreas. Over 90% of these patients have type 2 diabetes. In the past 40 years there has been a worldwide increase in diabetes prevalence. In 2000, the global prevalence of diabetes was approximately 2.8% and in 2014, it reached 8.3% according to the International Diabetes Federation (IDF). The results published by the Romanian Society of Diabetes, Nutrition, and Metabolic Diseases following completion of the PREDATORR study show that in 2014 in Romania there were nearly 2 million Romanians suffering from diabetes mellitus [1–3].

It might even be said that diabetes mellitus has become an epidemic disease that evolves in parallel with the aging population and increase in life expectancy, unhealthy lifestyle by transition to a Western-style diet (refined foods and saturated fats), sedentarism, and increasing prevalence of overweightness/obesity [4].

Type 2 diabetes mellitus (T2DM) and its micro- and macrovascular complications are recognized as a global public health problem due to their medical and socioeconomic costs. In general, T2DM is considered to be a multifactorial disease involving environmental factors, lifestyle, and genetic vulnerability. It is considered a heterogeneous syndrome characterized by chronic hyperglycemia and other metabolic alterations. T2DM's complications are both macro- and microvascular, the latter category including diseases such as neuropathy, nephropathy, and diabetic retinopathy [5, 6].

Diabetic neuropathy is a frequent complication of diabetes mellitus, its screening being justified by the serious consequences of this complication: ulceration and lower limb amputation at various levels and increased cardiovascular risk in case of autonomic nervous system neuropathy. It is estimated that diabetic neuropathy is present at the moment of type 2 diabetes mellitus diagnosis in about 10% of patients and affects about 50% of patients with a longer duration of the disease [7]. Diabetes mellitus can affect both the central and the peripheral somatic and autonomic nervous system structures, presenting with various clinical manifestations, the most common diabetic neuropathy manifestation being lower limbs Diabetic Sensorimotor Peripheral Neuropathy (DSPN), representing more than 90% of the total neuropathies in diabetes mellitus patients. As with other microvascular complications, risk factors include inadequate glycemic control, age, duration of diabetes, smoking, dyslipidemia, and hypertension, in particular diastolic blood pressure. Other independent risk factors appear to include height, obesity, presence of cardiovascular disease, presence of severe ketoacidosis, and presence of microalbuminuria. In the pathogenesis of diabetic neuropathy, both vascular mechanisms and nonvascular metabolic mechanisms are involved [6, 8, 9].

DSPN's etiology is multifactorial, some of the mechanisms that determine structural and functional nervous tissue damage being oxidative injuries, glucose metabolism polyol pathway activation, peripheral nerve storage of advanced glycation end products, and vascular insufficiency [7, 10]. Vascular endothelial cells are an important target for hyperglycemia-induced injury but the mechanisms that underlie these injuries are not fully understood. It has been suggested that in diabetes mellitus oxidative stress plays an important role in the pathogenesis of both microvascular and macrovascular complications. In diabetic patients, chronic hyperglycemia causes oxidative stress in tissues susceptible to complications [10, 11].

In diabetes mellitus, oxidative stress is associated with the appearance of neurons and glial support cells apoptosis, thus causing damage to the nervous system. Besides neuronal loss, in diabetes mellitus the regeneration ability is also impaired, particularly in the small diameter fibers. In patients with diabetic neuropathy, both degeneration and regeneration are simultaneously present, suggesting an intense dynamics of the disease. In time, the degeneration-regeneration balance shifts towards degeneration [12].

Oxidative stress occurs in a cellular system when the production of free radicals exceeds the antioxidant capacity of that system. In case of the cellular antioxidants' inability to remove free radicals, these attack and harm the proteins, lipids, and nucleic acids structures, resulting in various diseases such as diabetes mellitus and its complications, atherosclerosis, and cancer. The products oxidized or nitrosylated by free radicals have low biological activity, leading to energy metabolism losses, lowered transport cell signaling, and other major disorders. Accumulation of these injuries ultimately leads to cell death by necrosis or apoptosis [13].

A wide variety of factors contribute to variations in individual response to aggression factors, causing increased susceptibility to disease in certain individuals and increased resistance in others. Together with the development of molecular genetic technologies, investigations were directed towards DNA sequence variations in certain genes. Glutathione-S-transferases are phase II key detoxifying enzymes, which play an important role in cells protection against a wide variety of toxic insults caused by chemical products, metabolites, oxidative stress products, and electrophiles. They are involved in the conjugation with glutathione of a broad range of electrophilic substances, thus facilitating their detoxification, metabolism, and excretion. In humans at least eight families of related genes were found, alpha, mu, kappa, omega, pi, sigma, theta, and zeta encoded by* GSTA*,* GSTM*,* GSTK*,* GSTO*,* GSTP*,* GSTs*,* GSTT*, and* GSTZ* genes [14], genetic polymorphisms associated with low or altered enzyme activity being reported for* GSTM1*,* GSTT1*, and* GSTP1* [15], influencing the clearance of the DNA toxic intermediates, being partially responsible for the individual susceptibility of pancreatic beta cells and possibly the peripheral nerves to oxidative stress.

The most important gene polymorphism encodes a partial deletion of the* GSTM1* gene locus on chromosome 1p13.3 (*GSTM1* null genotype) which causes complete absence of enzyme activity.* GSTT1* gene, located on chromosome 22q11.2, presents a deletion similar to the* GSTM1* polymorphism. A substitution in the active site of valine with isoleucine at exon 5, codon 105 in the* GSTP1* gene 313, chromosome 11q13 leads to a Ile105Val amino acid substitution, this polymorphism leading to enzymatic activity deficiency and reduced ability to conjugate reactive electrophiles with glutathione, thus sensitizing the cells to damage mediated by free radicals [16, 17]. In case of deletions in* GSTM1* and* GSTT1* genes, a lack of enzyme activity is evidenced. In* GSTP1* polymorphism, the nucleotide transition of A 313 G leads to the Ile105Val substitution with the emergence of a new allele and the alteration of specific activities, compared to the wild type allele [18].

In our study we investigated several polymorphisms in* GST* genes and individual susceptibility to develop T2DM and also susceptibility of T2DM patients to develop DSPN. Thus, we conducted a case control study with a group consisting of a diabetic population with and without DSPN and a control group comprising a healthy control population. Both groups were genotyped for the* GSTM1*,* GSTT1*, and* GSTP1* polymorphisms that influence the detoxification of oxidative stress products. The aim of this study was to determine the frequency of GST genotypes in Romanian patients from the central area of the country with T2DM and without DSPN, compared to the control group and assessing the effect of these polymorphisms on the risk of T2DM and DSPN in patients with T2DM. To our knowledge, this study is the first to investigate the relationship of these polymorphisms with the appearance of DSPN in T2DM Romanian patients as several studies we have found offered different conclusions regarding the* GST* gene polymorphism and risk of T1DM and T2DM. One study we found investigated the* GSTM1* and* GSTT1* gene polymorphisms' involvement in cardiovascular autonomic neuropathy in adolescents with T1DM in Slovakia [19], but we failed to find any studies on the relationship between the polymorphisms of these genes and DSPN in T2DM.

## 2. Material and Method

### 2.1. Study Groups

This study was conducted in the university center of Tîrgu Mureș, Romania. Sampling was done from August 2014 to April 2015. Patients and controls were recruited from the neurology outpatients of the County Emergency Hospital affiliated to the University of Medicine and Pharmacy Tîrgu Mureș. In our case control study we included a total of 182 participants, including 84 unrelated patients with T2DM (59.5% women and 40.5% men) and an age-matched control group consisting of 98 unrelated individuals (53.1% women and 46.9% men) without T2DM, according to the medical history and laboratory tests. Patients were enrolled following the inclusion criteria: age over 18 years and diagnosis of T2DM, the exclusion criteria being history of blood disorders, endocrine disorders, known malignancies, kidney failure, liver failure, alcoholism or other possible causes of peripheral neuropathy, patients with diabetes secondary to chronic pancreatitis, Cushing's disease with treatment that can induce hyperglycemia, T1DM, and pregnant or lactating women. In the patients group, 50% of individuals were diagnosed with DSPN. The T2DM diagnosis was determined by a diabetologist according to the (revised) criteria of the American Association of Diabetology [20]. The diagnosis of DSPN was made by a neurologist through clinical exam and nerve conduction studies. All participants in this study gave their written informed consent for blood collection and biochemical and genetic analysis and for using their results in this report. The study protocol was approved by the Ethics Committee of the Clinical Emergency County Hospital Tîrgu Mureș and the Ethics Committee of the University of Medicine and Pharmacy of Tîrgu Mureș. All procedures were conducted according to the principles of the Helsinki Declaration.

The collected data included weight, height, duration of disease, systolic and diastolic blood pressure, presence of microalbuminuria, and type of administered hypoglycemic treatment. All patients were on oral hypoglycemic agents (OHA), on insulin therapy, or on combination therapy. Hypertension was considered in case of a systolic blood pressure value over 140 mmHg or a diastolic blood pressure value above 90 mmHg. The body mass index (BMI) was defined as weight in kilograms divided by the square of height in meters (kg/m^2^) and classified into four categories: underweight (BMI < 18.49), normal weight (BMI between 18.5 and 24.99), overweight (BMI between 25 and 29.99), and obese (BMI over 30). From each patient, two (2 mL on EDTA and 5 mL without anticoagulant) overnight fasting venous blood samples were collected. One sample was used for DNA extraction while the other served for biochemical analysis. From the latter one, HbA1c (glycated hemoglobin), total serum cholesterol, HDL cholesterol, triglycerides, urea, and creatinine were analyzed using a COBAS 6000 autoanalyzer, utilizing ROCHE methods and reagents. LDL cholesterol was calculated using Friedewald's formula, while microalbuminuria was analyzed from a 12-hour overnight urine sample.

### 2.2. DNA Extraction and* GST* Genotyping Analysis

Genomic DNA was isolated from the fresh collected blood samples using the Quick-gDNA MiniPrep kit (Zymo Research).

A multiplex polymerase chain reaction described by Sharma et al. was performed for genotyping the* GSTM1* and* GSTT1* gene polymorphisms [21]. In the case of* GSTP1* Ile105Val gene polymorphism, a polymerase chain reaction and restriction fragment length polymorphism (PCR-RFLP) method was performed using the primers, PCR protocol, and restriction enzyme as previously described by Hohaus et al. [22].

### 2.3. Statistical Analysis

Statistical analysis was performed in spreadsheets and GraphPad InStat 3 software, using a significance threshold alpha of 0.05. Data were considered as nominal or quantitative variables. Nominal variables were characterized using frequencies, while quantitative variables were tested for normality of distribution using Kolmogorov-Smirnov test and were characterized by median and percentiles (25–75%) or by mean and standard deviation (SD), when appropriate. A chi-square test was used in order to compare the frequencies of nominal variables. Quantitative variables were compared using *t*-test or Mann-Whitney test when appropriate. Deviations of allelic frequencies from the Hardy-Weinberg equilibrium were calculated using a chi-square test.

## 3. Results

Characteristics in both patients with T2DM (number = 84) and the control group (number = 98) are summarized in Table 1. Age distribution was not different in patients compared to controls (63.1 ± 8.9 and 60.8 ± 4.3, resp.) and neither was gender distribution. Prevalence distribution of* GSTM1*,* GSTT1*, and* GSTP1* genotypes in patients and controls is also presented in Table 1.* GSTP1* Val/Val genotype was significantly more frequent in T2DM patients than in controls (13.1% versus 5.1%), while* GSTP1* Ile/Ile genotype was more frequent in controls than in T2DM patients (73.5% versus 58.3%) (*p* = 0.05). Patients with T2DM and controls were not statistically different in terms of* GSTM1* or* GSTT1* genotypes.

*GSTM1* null and* GSTT1* null genotype frequencies were similar in both T2DM patients and controls. Regarding the* GSTP1* genotypes, Ile/Ile frequency in T2DM patients was 58.3% and 73.5% in controls and Ile/Val frequency was 28.6% in T2DM patients and 21.4% in controls while the frequency of Val/Val genotypes was 13.1% in diabetic patients and 5.1% in controls (*p* = 0.05).

In Tables 2 and 3 the frequency distributions of the double combination between* GSTM1* and* GSTT1* in patients and controls and in patients with T2DM, with or without DSPN, are presented. The combination of two genotypes showed no increased risk of developing T2DM or DSPN in diabetic patients, not even the combination of two high-risk genotypes,* GSTM1* null and* GSTT1* null. We found similar results for the combination between* GSTM1* and* GSTP1* and for* GSTT1* and* GSTP1* in patients and controls and in patients with T2DM, with or without DSPN.

Frequency distributions of the triple combination between* GSTM1*,* GSTT1*, and* GSTP1* in patients and controls and in patients with T2DM, with or without DSPN, are presented also in Tables 2 and 3. The combination of three genotypes showed no increased risk for developing T2DM or DSPN in diabetic patients.

Statistical analysis revealed that the* GSTP1* polymorphism is significantly associated with the risk of developing T2DM (*p* = 0.05) but was not associated with the risk of developing DSPN in diabetic patients.* GSTM1* and* GSTT1* gene polymorphisms were associated with neither the risk of developing T2DM nor the risk of DSPN occurrence in diabetic patients.

In Table 4 *GSTM1*,* GSTT1*, and* GSTP1* genotypes distributions between patients with T2DM and without DSPN and patients with T2DM and DSPN are shown, no evidence of a statistically significant difference between the two groups being found. Duration of disease (*p* = 0.0006), patient's age (*p* = 0.0001), elevated serum creatinine levels (*p* = 0.04), height (*p* = 0.04), high levels of microalbuminuria (*p* = 0.006), presence of insulin treatment (*p* = 0.02), and increased BMI (*p* = 0.05) were statistically significant, associated with the presence of DSPN (Table 4).

We performed correlations between two numeric variables (such as correlation between microalbuminuria and some parameter like HbA1, creatinine, duration of diabetes, blood urea nitrogen, HDL cholesterol, LDL cholesterol, total cholesterol, and triglycerides). We observed that microalbuminuria is significantly and negatively correlated only with HDL cholesterol and LDL cholesterol (correlation coefficient = −0.406, *p* < 0.0001, for HDL cholesterol and −0.445 for LDL cholesterol, *p* = 0.007) (Spearmen correlations).

In addition we performed a logistic regression regarding the presence of DSPN and different variables: total cholesterol, HDL cholesterol, LDL cholesterol, triglycerides, blood urea nitrogen and creatinine serum level, HbA1c, presence of microalbuminuria, age, duration of disease,* GSTM1*,* GSTT1*, and* GSTP1* polymorphisms, and insulin treatment. No statistical association was observed between the presence of DSPN and the variables previously mentioned (*p* > 0.05).

## 4. Discussions

The increased prevalence of T2DM is a major problem worldwide as well as in Romania, being a major healthcare burden through its treatment costs and related complications [2]. Diabetes is a chronic disease characterized by insufficient production of insulin or by an inability to effectively use the produced insulin, which leads to hyperglycemia and in time to the occurrence of serious damage to several of the body's systems and especially the nerves. Metabolic abnormalities within T2DM lead to mitochondrial superoxide overproduction, increasing the production of free radicals and intracellular reactive oxygen species (ROS), which in turn activate protein kinase (PKC) and increase the production of advanced glycation end products (AGEs). The imbalance between free radicals and antioxidant defense responses leads to oxidative stress, which may be a potential molecular mechanism involved in diabetic vascular complications [23].

GSTs are a group of multigenic and multifunctional enzymes involved in detoxification with a role in defending and protecting cells against chemical and metabolic insults as well as oxidative stress. The genes encoding the GST enzymes are polymorphic, therefore determining changes in the metabolic activity of each enzyme regarding the clearance of toxic DNA intermediates, and seem to determine the susceptibility of an individual's pancreatic *β* cells to oxidative stress and possibly the predisposition for T2DM's microvascular complications [23].* GSTM1* null and* GSTT1* null genotypes determine the enzymatic inactivity of the proteins encoded by these genes. Data published in recent years show that* GSTM1* and* GSTT1* null genotypes seem to be genetic risk factors for diseases such as T2DM and its cardiovascular complications [24]. In the present study, we investigated 3 major* GST* gene polymorphisms, M1, T1, and P1, in T2DM patients with and without DSPN and in healthy controls. The results of our present investigation showed a significant association between the frequency of* GSTP1* Val/Val genotype and T2DM. The* GSTM1* and* GSTT1* genotypes did not show any significant association with T2DM in our study population. We found no significant statistical association between* GSTM1*,* GSTT1*, and* GSTP1* genotypes and the presence of DSPN in patients with diabetes mellitus.

Data reported until now are conflicting regarding the association between* GSTP1* Ile105Val gene polymorphisms and the appearance of T2DM. There is no data reported on DSPN. Our results demonstrated that the frequency of the Val allele was higher in diabetics (27.4%) compared to the control group (15.8%) (OR = 2.0, 95% CI = 1.20–3.35, *p* = 0.007). In addition, significant differences in Val/Val genotype frequency in diabetic patients and controls (13.1% versus 5.1%, resp.) were observed. Our data suggest that Val allele of the* GSTP1* Ile105Val polymorphism and the* GSTP1* Val/Val genotype play an important role in individual susceptibility to T2DM but do not seem to influence the onset of DSPN in T2DM patients. Our data are consistent with data published by Amer et al. in 2012 and Bid et al., who showed that* GSTP1* Val allele and its variant genotype may help the emergence of T2DM [23, 25]. Ramprasath et al. also showed a significant risk for T2DM in patients harboring the* GSTP1* Ile/Val and Val/Val genotypes (OR = 1.423, 95% CI = 1.041–1.946, *p* = 0.027 and OR = 1.829, 95% CI = 1.064–3.142, *p* = 0.029) [26]. Data published by other researchers do not sustain the role of* GSTP1* Ile105Val gene polymorphisms in the appearance of T2DM, neither in Turkish nor in Iranian patients [15, 21, 27].

Our study also investigated the influence of* GSTM1* and* GSTT1* gene polymorphisms and the presence of T2DM (*p* = 0.96 and *p* = 0.21, resp.) besides the influence of* GSTM1* and* GSTT1* gene polymorphisms and susceptibility of diabetic patients to develop DSPN (*p* = 0.38 and *p* = 0.76, resp.). Genotype analysis did not show a clear association between studied single nucleotide polymorphisms (*GSTM1* and* GSTT1*) and the risk of diabetes mellitus or DSPN. The data obtained in our study were in accordance with the data published by Porojan et al. on a population from the center of Romania in whom increased frequencies of* GSTM1* null and* GSTT1* null genotypes in the T2DM group compared to controls were not observed (*p* = 0.171, OR = 1.444, 95% CI = 0.852–2.447; *p* = 0.647, OR = 0.854, 95% CI = 0.436–1.673). The combined* GSTM1*/*GSTT1* null genotypes were not statistically significant, higher in our T2DM patients compared to controls (*p* = 0.67, OR = 1.82, 95% CI = 0.32–2.08), in contrast with the results of the previously mentioned study (*p* = 0.0021, OR = 0.313, 95% CI = 0.149–0.655) [28].

In their study Yalin et al. found that patients with T2DM had a higher* GSTM1* null genotype frequency than the control group (OR = 3.7, 95% CI = 2.5–6.70) [27]. Another Turkish study reported similar data regarding the influence of* GSTM1* null allele on susceptibility to T2DM, with a higher frequency of the* GSTM1* null genotype compared to that in the control group (OR = 3.7, 95% CI = 2.05–6.70) [15]. Ramprasath et al. in a South Indian population found that the* GSTM1* null genotype was associated with a two-time increased risk of T2DM appearance (OR = 2.925, 95% CI = 2.078–4.119, *p* = 0.0001) [26]. Wang et al. in a study on a Chinese population found no significant differences between patients with T2DM and controls regarding the frequency of* GSTM1 *null genotype (OR = 1.18, 95% CI = 0.34–4.16, *p* > 0.05) [29], results that are in agreement with our findings.

Similar to our results, no significant effect on the risk of T2DM appearance was observed regarding the* GSTT1* null/present gene polymorphisms either by Yalin et al. [27] or by Gönül et al. [15] in Turkish patients. Ramprasath et al. in a South Indian population found that the* GSTT1* null genotype was associated with a three-time increased risk of T2DM appearance (OR = 3.114, 95% CI = 2.176–4.456, *p* = 0.0001) [15, 26]. Wang et al. in their study found that the* GSTT1* present genotype was associated with a significant decrease of T2DM appearance in comparison to the null genotype [29].

We also investigated the combined effect of variant genotypes as it might have a synergic effect in comparison with the individual genotypes. Firstly, we investigated the combined effect of these polymorphisms and the presence of DSPN in T2DM. Neither single nucleotide polymorphism (SNP) (*GSTM1* and* GSTT1*) nor the two combined or even the three combined genotypes (*GSTP1* Ile105Val,* GSTT1*, and* GSTM1*) affected the risk of T2DM or DSPN occurrence in our patients. Gönül et al. in Turkish patients found that carriers of combined* GSTM1* null and* GSTT1* null, associated with a complete lack of enzyme activity, and* GSTP1* Ile/Val genotype had an increased risk of developing T2DM (OR = 4.118, 95% CI = 1.327–12.778, *p* = 0.009) [15].

Our results regarding* GSTM1* genotype are not consistent with most of the cited studies which might be due to the small number of cases and the impact of ethnicity on general characteristics of investigated patients.

The present study suggests that* GSTP1* genes may contribute to the development of T2DM in Romanian population. There is only one study, published in 2015, referring the* GSTM1* and* GSTT1* polymorphisms in a Romanian T2DM population [28] but according to our knowledge no other studies in Romania or in other countries investigated the effect of these polymorphisms in T2DM DSPN. In the medical literature we found only one study on* GSTM1* and* GSTT1* gene polymorphisms and cardiovascular autonomic neuropathy (CAN) in T1DM in Slovak adolescents [30] but we did not find studies regarding these gene polymorphisms in T2DM DSPN. This is the first study investigating the association between the combined effect of* GSTM1*,* GSTT1*, and* GSTP1* genotypes in a population consisting of a control group and T2DM patients with and without DSPN in Romania and to our knowledge the first study of its kind to investigate the effect of these combined genotypes on the occurrence of DSPN in T2DM.

Unlike other diseases such as neoplasms [31, 32] in which* GSTM1* and* GSTT1* polymorphisms are extensively investigated, there are few studies that investigate the association between* GSTM1*,* GSTT1*, and* GSTP1* gene variants and the presence of microvascular complications in T1DM and T2DM [33, 34]. A Slovenian study published in 2012 found that the* GSTT1* null genotype was associated with an increased risk of diabetic retinopathy (DR) in T2DM (OR = 2.303, 95% CI = 1.649–3.216, *p* < 0.001) while the* GSTM1* null genotype conferred a reduced risk of T2DM DR (OR = 0.475, 95% CI = 0.339–0.668, *p* < 0.001). There was no evidence that the* GSTP1* gene variants were associated with DR in their study [34]. Datta et al. reported that the* GSTT1* null genotype was associated with diabetic nephropathy [35]. Dadbinpour et al. reported no significant relationship between* GSTT1* genotype and the presence of DR in T2DM (*p* = 0.187) but reported a significant association between* GSTM1* genotype and DR (*p* = 0.04), results in contradiction with a study highlighting that GSTM1 null genotype might confer protection against DR in Caucasians with T2DM [36]. An Iranian study showed that the presence of* GSTM1* null genotype and the synergic effect of* GSTM1* null and* GSTT1* null genotypes were associated with an increased risk of T2DM development but there was no significant correlation observed between* GSTM1* null and* GSTT1* null genotypes and the risk of DR occurrence [37]. Doney et al. demonstrated that* GSTT1* null genotype is associated with a generalized vasculopathy and an increased risk for progression to DR and diabetic nephropathy [24]. A study that included patients with T1DM found out that* GSTM1* present-type genotype was significantly more frequent in patients with DR but found no significant difference in allele and genotype frequencies for* GSTT1* polymorphisms in association with DR or diabetic nephropathy. Also, the carriers of both* GSTM1* null and* GSTT1* null genotypes, resulting in a complete lack of enzyme activity, did not have an increased risk for developing DR or diabetic nephropathy [33]. These results are similar with our DSPN results.

The only study we found that investigated* GSTM1* and* GSTT1* gene polymorphisms and the nervous system was in T1DM Slovak adolescents with cardiovascular autonomic neuropathy (CAN). Their results showed that* GST* genes polymorphisms may play a partial role in the pathogenesis of CAN in patients with T1DM.* GSTT1* present-type genotype and* GSTT1/M1* present/null combination were associated with CAN [30].

The conflicting data obtained from genetic studies on the influence of* GST* genes polymorphisms on microvascular complications of T2DM and the lack of data on polymorphisms of these genes and DSPN leave room for further research in order to establish the role of these polymorphisms in the development of microvascular complications of T2DM, including the risk of DSPN occurrence.

In the present study we also investigated several clinical and biochemical parameters in patients with T2DM with and without DSPN: patient's age, onset age of T2DM, duration of diabetes, BMI, HbA1c level, lipid profile, urea, creatinine, systolic and diastolic blood pressure, lipid-lowering treatment, and insulin administration. The most remarkable results we found were for age (*p* = 0.0001), duration of diabetes (*p* = 0.0006), height (*p* = 0.04), BMI (*p* = 0.05), creatinine (*p* = 0.04), microalbuminuria (*p* = 0.006), and the presence of insulin treatment (*p* = 0.02) which were statistically significant, associated with the presence of DSPN.

Tesfaye et al. studied 3250 diabetic patients and reported a correlation of diabetic polyneuropathy with advanced age [38] and our data show a statistically significant difference, advanced age and duration of diabetes being correlated with the presence of DSPN. In interpreting the results we must take into account that most often patients had an advanced age and therefore a longer evolution of the disease.

Taller persons are more likely to develop DSPN because they have longer peripheral nerves. Our results are consistent with clinical studies on patients with T2DM, which show that height is associated with a higher frequency of nerve dysfunction including decreased nerve conduction velocity and vibration perception threshold [39]. Patients with DSPN present severe axonal loss at distal level, the neuropathy being initially evident in the distal lower limb, probably due to the greater axons length in the legs. A higher nerve length increases the vulnerability towards metabolic and transport dysfunctions [40]. Increased height is associated with an elevated pressure on the lower extremities which leads to decreased capillary blood flow [38, 41].

Not many studies have focused on the role of obesity in the development of microvascular complications. There are studies which reported no association between BMI and the presence of DSPN [41, 42] and others that have not even considered obesity when the involved risk factors were assessed in DSPN etiopathogenesis [43, 44]. Pirart's study is the first which reported that DSPN prevalence is increased in obese patients compared to those with normal weight in the first 15 years of the onset of diabetes mellitus [45], the presented data being in correlation with the results of our study.

Important data derived from the Rochester cohort longitudinal assessment, in which Dyck et al. reported that glycated hemoglobin, duration of diabetes, and type of diabetes were all independent risk factors for the appearance of polyneuropathy while severity of retinopathy, renal insufficiency, and proteinuria were important covariates [46]. In our patients creatinine levels and the presence of microalbuminuria were correlated with the presence of DSPN. In the EURODIAB IDDM Complications Study, positive correlations were observed between the presence of DSPN and advanced age, height, and duration of diabetes but were observed also with high blood diastolic pressure, hypertriglyceridemia, and presence of microalbuminuria [47], part of these observations being also confirmed by our study.

Independent of genetic factors, insulin therapy was associated with DSPN. This effect could be explained by differences in hypoglycemia frequency between the two groups. The association between DSPN and insulin therapy may be due to an adverse effect of insulin administration, but more likely this association occurs because insulin therapy is being administrated in advanced T2DM stages, when the incidence of microvascular complications, including DSPN, is also higher.

Our study has several limitations. Firstly, the small number of patients is a major limitation and the obtained data may not be conclusive for the general population. Further investigations in large scale cohort studies in different ethnic groups are needed in order to establish the role of* GSTM1*,* GSTT1,* and* GSTP1* gene polymorphisms in the pathogenesis of T2DM and its microvascular complications such as DSPN. Another limitation of our study was that only several polymorphisms in the* GST* gene have been investigated. Other studies on genes involved in ROS production/elimination such as manganese superoxide dismutase (*MnSOD*), catalase (*CAT*), and glutathione-peroxidase-1 (*GPX1*) are recommended. However, despite small number of patients included our study, it still is valuable being the first one to investigate* GSTM1*,* GSTT1,* and* GSTP1* SNPs and their combined effect on microvascular complications such as DSPN in T2DM.

## 5. Conclusions

In conclusion, the present study suggests that* GSTP1* gene polymorphisms may contribute to the development of T2DM in Romanian population. Our data suggest that* GSTM1*,* GSTT1*, and* GSTP1* gene polymorphisms are not associated with individual susceptibility to developing DSPN in patients with T2DM.

## Figures and Tables

**Table 1:** Baseline characteristics of studied/control patients.

Variable	T2DM, No. (%)	Controls, No. (%)	*p *value
Patients number	84	98	
Male/female	34 (40.5)/50 (59.5)	46 (46.9)/52 (53.1)	0.38
Age (years)	63.1 ± 8.9	60.8 ± 4.3	0.65
*GSTP1*			
Ile/Ile	49 (58.3)	72 (73.5)	0.05
Ile/Val	24 (28.6)	21 (21.4)	
Val/Val	11 (13.1)	5 (5.1)	
*GSTT1*			
Null	15 (17.9)	25 (25.5)	0.21
Present	69 (82.1)	73 (74.5)	
*GSTM1*			
Null	40 (47.6)	47 (48.0)	0.96
Present	44 (52.4)	51 (52.0)	

No.: number.

**Table 2:** Genetic characteristics of studied patients with T2DM and controls.

Variable	T2DM-84, No. (%)	Controls-98, No. (%)	*p *value
*GSTM1*/*GSTT1*	36 (42.8)	35 (35.7)	Reference
*GSTM1 *null/*GSTT1* null	11 (13.1)	13 (13.3)	0.67, OR: 1.82 95% CI: (0.32–2.08)
*GSTM1* present/*GSTT1* null	4 (4.7)	12 (12.2)	0.09, OR: 0.32 95% CI: (0.09–1.10)
*GSTM1 *null/*GSTT1 *present	33 (39.3)	38 (38.7)	0.61, OR: 0.84 95% CI: (0.43–1.63)
*GSTP1/GSTT1/GSTM1*			
Ile/Ile/present/present	21 (25.0)	24 (24.5)	Reference
Ile/Ile/null/null	6 (7.1)	12 (12.2)	0.40, OR: 0.57 95% CI: (0.18–1.79)
Ile/Ile/null/present	3 (3.6)	8 (8.2)	0.31, OR: 0.42 95% CI: (0.10–1.82)
Ile/Ile/present/null	19 (22.6)	28 (28.6)	0.54, OR: 0.77 95% CI: (0.34–1.77)
Ile/Val/null/null	3 (3.6)	1 (1.02)	0.34, OR: 3.43 95% CI: (0.33–35.5)
Ile/Val/null/present	0 (0.0)	3 (3.06)	—
Ile/Val/present/null	10 (11.9)	7 (7.14)	0.57, OR: 1.63 95% CI: (0.52–5.05)
Ile/Val/present/present	11 (13.1)	10 (10.2)	0.79, OR: 1.25 95% CI: (0.44–3.55)
Val/Val/null/null	2 (2.4)	0 (0.0)	—
Val/Val/null/present	1 (1.2)	1 (1.02)	—
Val/Val/present/null	4 (4.8)	3 (3.06)	0.69, OR: 1.52 95% CI: (0.30–7.6)
Val/Val/present/present	4 (4.8)	1 (1.02)	0.34, OR: 4.57 95% CI: (0.47–44.2)

OR: odd ratio, CI: confidence interval, and No.: number.

**Table 3:** Genetic characteristics of studied patients with T2DM.

Variable	DSPN No-42, No. (%)	DSPN Yes-42, No. (%)	*p *value
*GSTM1* present/*GSTT1* present	16 (38.1)	20 (47.1)	Reference
*GSTM1* null/*GSTT1* null	5 (11.9)	6 (14.3)	0.73, OR: 0.66 95% CI: (0.17–2.59)
*GSTM1* present/*GSTT1 *null	2 (4.7)	2 (4.7)	0.95, OR: 0.80 95% CI: (0.10–6.32)
*GSTM1* null/*GSTT1* present	19 (45.2)	14 (33.3)	0.27, OR: 0.58 95% CI: (0.22–1.53)
*GSTP1/GSTT1*/*GSTM1*			
Ile/Ile/ present/present	13 (30.9)	8 (19.1)	Reference
Ile/Ile/ null/null	4 (9.5)	2 (4.7)	0.98, OR: 0.81 95% CI: (0.12–5.5)
Ile/Ile/ null/present	2 (4.8)	1 (2.4)	0.98, OR: 0.81 95% CI: (0.06–10.48)
Ile/Ile/ present/null	7 (16.6)	12 (28.5)	0.11, OR: 2.78 95% CI: (0.77–10.1)
Ile/Val/null/null	0 (0.0)	2 (4.8)	0.17, OR: 7.9 95% CI: (0.33–186.4)
Ile/Val/null/present	0 (0.0)	0 (0.0)	—
Ile/Val/present/null	5 (11.9)	6 (14.3)	0.46, OR: 1.95 95% CI: (0.44–8.55)
Ile/Val/present/present	6 (14.2)	5 (11.9)	0.72, OR: 1.35 95% CI: (0.30–5.94)
Val/Val/null/null	1 (2.4)	1 (2.4)	—
Val/Val/null/present	0 (0.0)	1 (2.4)	—
Val/Val/present/null	2 (4.8)	2 (4.8)	0.95, OR: 1.62 95% CI: (0.19–13.9)
Val/Val/present/present	0 (0.0)	3 (7.2)	0.08, OR: 11.1 95% CI: (0.51–243.3)

OR: odd ratio, CI: confidence interval, No.: number. DSPN No: without DSPN, and DSPN Yes: with DSPN.

**Table 4:** Baseline characteristics of studied patients with T2DM.

Variable	DSPN No	DSPN Yes	*p *value
Patients number	42	42	
Male/female, No. (%)	23 (54.8)/19 (45.2)	27 (64.3)/15 (35.7)	0.34
Age (years)	60.1 ± 7.5	66.2 ± 9.3	0.0001
Height m^2^	1.65 ± 0.06	1.68 ± 0.07	0.04
Weight kg	85.4 ± 13.8	83.0 ± 12.7	0.40
Age at diabetes diagnosis (years)	53.7 ± 8.5	55.5 ± 10.8	0.38
Diabetes duration (years)^*∗*^	5.5 (1–17)	10 (1–29)	0.0006
*GSTP1*, No. (%)			
Ile/Ile	26 (61.9)	23 (54.8)	0.68
Ile/Val	12 (28.6)	12 (28.6)	
Val/Val	4 (9.5)	7 (16.7)	
*GSTT1*, No. (%)			
Null	8 (19.0)	7 (16.7)	0.76
Present	34 (81.0)	35 (83.3)	
*GSTM1*, No. (%)			
Null	22 (52.4)	18 (42.9)	0.38
Present	20 (47.6)	24 (57.1)	
Body mass index (kg/m^2^)	29.2 ± 4.3	31.1 ± 5.1	0.05
Systolic BP (mmHg)	152.5 ± 22.2	154.2 ± 28.4	0.75
Diastolic BP (mmHg)	89.1 ± 9.8	90.5 ± 17.1	0.62
Hypertension (yes), No. (%)	40 (95.2)	41 (97.6)	0.55
Creatinine (mg/dL)	0.87 ± 0.18	0.94 ± 0.28	0.04
Blood urea nitrogen (mg/dL)	36.8 ± 12.6	42.3 ± 15.7	0.08
HDL cholesterol (mg/dL)	43.5 ± 11.8	41.8 ± 12.2	0.52
LDL cholesterol (mg/dL)	110.3 ± 33.5	108.5 ± 52.2	0.84
Total cholesterol (mg/dL)	184.7 ± 40.7	184.3 ± 63.3	0.97
Triglycerides (mg %)^*∗*^	142.4 (70–286.1)	153.1 (59.1–798.0)	0.92
Microalbuminuria (mg/L)^*∗*^	3.9 (3–234.1)	13.5 (3–1293)	0.006
HbA1c	6.95 ± 1.01	7.3 ± 1.2	0.24
Statins (yes), No. (%)	24 (57.1)	30 (71.4)	0.17
Fibrates (yes), No. (%)	1 (2.4)	1 (2.4)	0.98
Insulin (yes), No. (%)	13 (31.0)	23 (54.8)	0.02

Data are shown as mean ± SD, as median (min-max), or as OR: odd ratio, CI: confidence interval, and No.: number.

^*∗*^Mann Whitney test, DSPN No: without DSPN, and DSPN Yes: with DSPN.
